# Problem gambling and affected others: health, relational and economic outcomes across longitudinal trajectories in a population-based Swedish study

**DOI:** 10.1186/s12889-026-28778-z

**Published:** 2026-08-10

**Authors:** Kristina Sundqvist, Peter Wennberg

**Affiliations:** 1 Department of Psychology, Stockholm University, Stockholm, Sweden; 2 Department of Public Health Sciences, Stockholm University, Stockholm, Sweden; 3 Department of Psychology, University of Inland, Lillehammer, Norway; 4 Department of Global Public Health, Karolinska Institutet, Stockholm, Sweden

**Keywords:** Affected others, Problem gambling, Longitudinal study, Exposure trajectories, Gambling-related harm

## Abstract

**Background:**

Exposure to another person’s gambling problems has been linked to a range of adverse outcomes among affected others (AOs); however, most evidence is based on cross-sectional studies. Less is known about how such outcomes vary over time and across different patterns of exposure.

**Aim:**

This study examined health (including mental distress, suicidal ideation, hazardous alcohol use, and own gambling behaviour), relational (arguing, exposure to violence), and economic outcomes longitudinally across different trajectories of AO exposure in the general population.

**Methods:**

Data were drawn from two waves (2018 and 2021) of the Swedish Longitudinal Gambling Study (Swelogs). The analytic sample included 2 814 respondents who participated in both waves. AO status was based on a single item asking whether the respondent knew someone close to them who, in their own opinion, had problems with gambling in the past 12 months, and was classified into four trajectories: never AO, former AO, newly AO, and persistent AO. Outcomes in 2021 were compared between each AO trajectory and never AO respondents using chi-square tests and logistic regression models, adjusting for sex, age, and own gambling.

**Results:**

Persistent AOs showed consistently higher prevalence and odds of adverse outcomes across all domains, including serious mental distress, hazardous alcohol use, relational conflict, violence exposure, and economic hardship. Former AOs generally showed attenuated risks relative to persistent AOs, although economic hardship remained significantly elevated after adjustment. Newly AOs exhibited elevated odds of hazardous alcohol use, increased arguing, and economic hardship compared to never AOs.

**Conclusions:**

The findings suggest that AO status may be better conceptualised as a dynamic condition than a fixed one. Both sustained and past exposure to another person’s gambling problems were associated with adverse outcomes, with economic hardship showing a particularly persistent pattern. Prevention and support initiatives should consider not only current exposure but also exposure history when identifying individuals at risk. In addition, given the consistent associations observed between AO status and hazardous alcohol use, relational conflict, and economic hardship, individuals presenting with these difficulties in clinical or support settings may also benefit from being asked about exposure to a close other’s gambling problems.

**Supplementary information:**

The online version contains supplementary material available at 10.1186/s12889-026-28778-z.

## Introduction

Exposure to another person’s gambling problems has been linked to substantial consequences for those in their social environment, commonly referred to as affected others (AOs). These consequences span multiple domains of everyday life, including mental health, social relationships, and financial stability. Studies indicate that AOs face elevated risks of psychological distress, depression, and suicidality, as well as increased exposure to interpersonal conflict, intimate partner violence, and economic hardship [1–3]. Exposure to a close other’s gambling problems has also been linked to elevated alcohol use [4, 5] and an increased likelihood of developing gambling problems of one’s own [6]. Financial harm, such as difficulties in managing everyday expenses or debt accumulation, appears particularly persistent and may be closely linked to the duration and timing of exposure [7]. Qualitative accounts further describe how affected others experience constrained day-to-day spending, debt accumulation, and long-term financial insecurity as a consequence of a close other’s gambling [8].

Estimates from population surveys indicate that the number of affected others substantially exceeds the number of individuals with gambling problems. Each individual with a gambling problem is estimated to adversely affect six others [9], and between 4.5% nd over 20% of adults in the general population report being negatively affected by someone else’s gambling, depending on definitions and measurement approaches [10–12]. In Sweden, a recent population-based study found that approximately 11% of adults reported having someone with gambling problems in their close social environment during the past year [13]. This exposure is not limited to partners or household members but extends across broader social networks, including family members, friends, and other close social ties [4, 13–16].

A key limitation of the existing literature is that most studies rely on cross-sectional designs, which provide limited insight into how these consequences develop or change over time. From a theoretical perspective, the duration and continuity of exposure may be central to understanding harm, as sustained exposure to a close other’s problematic behaviour is likely to involve ongoing stress and cumulative strain over time, whereas the cessation of exposure may allow for some degree of recovery [17]. This distinction suggests that adverse outcomes may not only reflect the presence of exposure but also its temporal pattern. It remains unclear whether the adverse outcomes observed among AOs primarily reflect short-term responses to acute exposure or more enduring effects associated with prolonged or repeated exposure. Clarifying these temporal dynamics is essential for understanding whether harm diminishes when exposure ends or persists even after the gambling problems of the affected person have been resolved.

Longitudinal evidence on affected others remains relatively scarce. The few available studies suggest that some outcomes, such as relational conflict and psychological distress, may improve following the cessation of exposure [5], whereas longitudinal evidence indicates that social and financial outcomes are closely linked to the timing of exposure [7]. Using an eight-wave longitudinal design, Kauppila et al. [6] further found that exposure to a family member’s or friend’s gambling problems increased affected others’ own risk of developing problem gambling, with strong family relationships, but not friendships, buffering against this risk.

However, existing longitudinal research has largely focused on overall changes over time and has given less attention to how variations in exposure are linked to outcomes. Taken together, these limitations make it difficult to determine how adverse outcomes relate to sustained exposure, whether they persist after exposure has ended, or how they respond to changes in exposure conditions.

Against this background, longitudinal population-based studies that explicitly distinguish between different patterns of AO exposure over time are needed. Conceptualising AO status as a dynamic rather than static condition, and examining how outcomes vary across distinct exposure trajectories, may provide important insights into the persistence, accumulation, and potential reversibility of gambling-related consequences among affected others.

### Aim and research question

This study examined health (mental distress, suicidal ideation, hazardous alcohol use, and own gambling behaviour), relational (arguing, exposure to violence), and economic outcomes longitudinally across different trajectories of affected others in a population-based Swedish sample. Specifically, the study addressed the following research question: What are the patterns of health, relational, and economic outcomes among individuals classified as former, newly, and persistent affected others compared to never AOs over a three-year period?

## Methods

### Participants

This study uses the two most recent waves of the Swedish Longitudinal Gambling Study (Swelogs), conducted by the Public Health Agency of Sweden. In 2015, a sample of 21 000 individuals aged 16–84 was selected, of whom approximately 9 400 responded and were re-contacted in 2018 alongside a new sample of approximately 4 000 individuals. The present study is based on respondents who participated in both the 2018 and 2021 waves of the survey. The 2015 wave was not included in the analyses because the new sample added in 2018 had no baseline data from 2015, which would have required either excluding these respondents or handling substantial missing data at baseline.

The 2018 cohort consisted of 5 080 participants and they were re-contacted in 2021. The follow-up study had 3 005 respondents, corresponding to a response rate of 59%.

Of the 3 005 respondents who participated in both the 2018 and 2021 survey waves, 191 were excluded due to missing responses to the affected-other question at one or both time points, resulting in an analytic sample of 2 814 respondents who provided valid responses to the affected-other question at both time points. Participants were classified as never AO (*n* = 2 416), former AO (*n* = 144), newly AO (*n* = 154), or persistent AO (*n* = 100). The baseline sociodemographic characteristics according to AO status are presented in Table 1.

**Table 1:** Socio-demographic characteristics of the sample at baseline (2018) across AO status group in 2021. Percentages and (n)

	Characteristic	Never AO (*n* = 2 416)	Former (*n* = 144)	Newly (*n* = 154)	Persistent (*n* = 100)
Gender	Women, % (*n*)	54.5 (1 316)	62.5 (90)	45.5 (70)	52.0 (52)
Age groups	16–24 years	23.9 (578)	29.9 (43)	50.0 (77)	43.0 (43)
	25–44 years	15.5 (374)	21.5 (31)	13.0 (20)	22.0 (22)
	45–64 years	31.0 (749)	30.6 (44)	23.4 (36)	26.0 (26)
	≥ 65 years	29.5 (713)	18.1 (26)	13.6 (21)	9.0 (9)
Education	Pre-secondary education	17.7 (427)	16.0 (23)	29.2 (45)	23.0 (23)
	Upper secondary education	36.9 (891)	46.5 (67)	38.3 (59)	44.0 (44)
	Post-secondary education	44.7 (1 078)	37.5 (54)	31.8 (49)	33.0 (33)

### Measures

#### Affected other status

AO status was measured using a single question: Is there anyone close to you who, in your opinion, has had problems with gambling during the past 12 months? The response options were “No,” “Yes, one person,” and “Yes, several persons,” with the latter two combined into a single affirmative category in the analyses. Hence, AO status reflects exposure to someone with gambling problems in the close social environment rather than directly experienced harm, and represents a broad operational definition consistent with previous population-based studies [5, 18, 19].

#### Socio-demographic variables

Sociodemographic variables included age (16–24, 25–44, 45–64, and ≥ 65 years), as reported in the survey, as well as sex (male and female) and education (less than upper secondary, upper secondary, and post-secondary), which were obtained from national population registers.

#### Health outcomes

##### Serious mental distress

Was assessed using the Kessler Psychological Distress Scale (K6; [20]), referring to the past 30 days. The K6 consists of six items asking respondents how often they felt: (1) nervous, (2) hopeless, (3) restless or fidgety, (4) so depressed that nothing could cheer them up, (5) that everything was an effort, and (6) worthless. Response options ranged from never to all of the time and were coded as 0–4. The item scores were summed to create a total score, with higher scores indicating greater psychological distress. Established cut-offs were used to identify serious (13+) mental distress [21].

##### Past-year suicidal ideation

was assessed using a single item asking respondents whether they had experienced thoughts of taking their own lives during the past 12 months (yes/no). This question was not asked in 2018; hence, it was only used in the descriptives of health among AOs in 2021.

Gambling outcomes were assessed using the Problem Gambling Severity Index (PGSI; [22]). Two thresholds were applied: a score of 1 or higher indicating *at-risk gambling*, and a score of 3 or higher indicating *moderate-risk gambling*. In the adjusted models examining non-gambling outcomes, moderate-risk gambling was included as a covariate. Only respondents who reported having gambled in the past 12 months were administered the PGSI items; those who had not gambled were coded as 0 on the PGSI.

##### Hazardous alcohol use

Was measured using the brief questionnaire Alcohol Use Disorders Identification Test—Consumption (AUDIT-C; [23]), applying cut-off scores of ≥ 5 for women and ≥ 6 for men.

#### Relational outcomes

##### Increased arguing

Was measured by asking whether respondents had argued more with someone close to them in the past 12 months than before. Exposure to physical *violence* or threats of violence during the past year was assessed using a single item asking whether the respondent had been subjected to physical violence or threats of violence that caused fear during the past 12 months (yes/no).

#### Economic outcomes

##### Economic hardship

Was assessed by asking whether respondents had experienced difficulties managing everyday expenses such as food, rent, and bills during the past 12 months; the response options “Yes, on one occasion” and “Yes, on several occasions” were combined into a single affirmative category, while “Don’t know” and “Prefer not to answer” were coded as missing.

Sample sizes varied across outcomes due to item-specific missing data. PGSI scores had no missing data (*N* = 2 814), whereas the sample sizes for other outcomes varied: alcohol use (*N* = 2 809), serious mental distress (*N* = 2 746), increased arguing (*N* = 2 649), violence (*N* = 2 796), suicidal ideation (*N* = 2 797), and economic hardship (*N* = 2 776).

### Analyses

Analyses were based on respondents who participated in both the 2018 and 2021 survey waves. Affected-other (AO) status was classified longitudinally into four mutually exclusive groups: never AO, former AO (only in 2018), newly AO (only in 2021), and persistent AO (at both time points).

Descriptive statistics were used to summarise baseline sociodemographic characteristics and the prevalence of outcomes in 2021 across AO groups. Differences in the prevalence of health, relational, and economic outcomes between each AO group and never affected respondents were assessed using chi-square tests; statistical significance is indicated in the tables using asterisks.

Changes in outcomes between 2018 and 2021 were examined descriptively and illustrated graphically to visualise temporal patterns within each AO group. These analyses were intended to describe trends over time rather than to support causal inferences. Within-group changes in outcomes between 2018 and 2021 were separately examined within each AO trajectory. For continuous index measures (alcohol use, gambling severity, and mental distress), Wilcoxon signed-rank tests were used because of non-normal distributions and a high proportion of zero values. For binary outcomes (increased arguing, exposure to violence, and economic hardship), within-group changes were assessed using McNemar’s test. When the number of discordant pairs was small, exact p-values based on binomial distribution were reported. All within-group analyses were based on paired observations, resulting in outcome-specific sample sizes.

The associations between AO status and outcomes in 2021 were further examined using logistic regression models. Results are presented as odds ratios (ORs) with 95% confidence intervals (CIs), with never AO respondents as the reference group. Crude models were estimated for each outcome, followed by adjusted models including sex, age group, and moderate-risk gambling. In the adjusted models, female sex, the oldest age group (≥ 65 years), and not at-risk gambling were used as reference categories. Own moderate-risk gambling (PGSI ≥ 3) was included as a covariate in all adjusted models, except for the at-risk gambling outcome.

All outcomes were analysed as binary variables. Responses coded as “don’t know” or “prefer not to answer” were treated as missing data. All analyses were conducted using IBM SPSS Statistics (version 29), and statistical significance was set at *p* < 0.05.

No adjustment for multiple comparisons was applied, as this study was exploratory in nature, aiming to describe patterns of association across a range of outcomes rather than to test a small number of pre-specified confirmatory hypotheses. This approach follows recommendations for exploratory research, where overly conservative correction procedures risk obscuring meaningful patterns at the cost of increased type II error [19]. We acknowledge that this increases the probability of false-positive findings, and results should therefore be interpreted as hypothesis-generating rather than confirmatory.

## Results

### Prevalence of adverse outcomes in 2021 by affected other trajectory

Marked differences in outcomes were observed in 2021 across the longitudinal AO trajectories (Table 2). Individuals with persistent affected-other exposure consistently reported the highest prevalence of adverse outcomes. For example, past year serious mental distress was reported by 15.0% of persistent AOs (corresponding to *n* = 15), compared with 6.1% among never affected respondents (corresponding to *n* = 147). Similarly, suicidal ideation during the past 12 months was reported by 11.0% of persistent AOs versus 2.6% among never affected respondents (Table 2).

**Table 2:** Prevalence of past year health, relational, and economic outcomes in 2021 by longitudinal affected-other status. Percentages and 95% confidence intervals. Pairwise χ² tests comparing each affected-other trajectory with never affected respondents

		Never AO (*n* = 2 416)	Former AO (*n* = 144)	Newly AO (*n* = 154)	Persistent AO (*n* = 100)
Mental Health	Serious mental distress (Kessler ≥ 13)	6.1 (5.2–7.2)	9.7 (5.9–15.7)	7.1 (4.0–12.3)	15.0 (9.3–23.3)***
	Suicidal ideation	2.6 (2.1–3.3)	4.9 (2.4–9.7)	4.5 (2.2–9.1)	11.0 (6.3–18.6)***
	Hazardous alcohol use (AUDIT-C 5/6+)	13.3 (12.0–14.7)	19.4 (13.8–26.7)*	24.7 (18.5–32.1)***	26.0 (18.4–35.4)***
	At-risk gambling (PGSI 1+)	2.7 (2.2–3.5)	5.6 (2.8–10.6)	4.5 (2.2–9.1)	11.0 (6.3–18.6)***
	Moderate-risk Gambling (PGSI 3+)	0.7 (0.4–1.1)	1.4 (0.39–5.0)	1.3 (0.4–4.7)	7.5 (3.7–14.7)***
Relational outcomes	Increased arguing with close others	7.7 (6.7–8.9)	12.0 (7.5–18.7)	16.8 (11.6–23.8)***	19.2 (12.5–28.3)***
	Violence	1.4 (1.0–1.9)	4.2 (1.9–8.9)**	3.9 (1.8–8.2)*	5.1 (2.2–11.3)**
Economic outcome	Difficulties managing everyday expenses	6.3 (5.4–7.3)	14.6 (9.7–21.2)***	15.3 (10.4–22.0)***	17.4 (11.1–26.0)***

*p < 0.05; ** *p* < 0.01; *** *p* < 0.001

Hazardous alcohol use was also more prevalent among affected others, increasing from 13.3% among never affected respondents to 26.0% among persistent AOs. At-risk gambling (PGSI ≥ 1) was reported by 11.0% of persistent affected others compared with 2.7% among never affected respondents. Elevated prevalence was likewise observed for relational and economic outcomes: increased arguing with close others was reported by 19.1% of persistent AOs compared with 7.7% among never affected respondents, while economic hardship was reported by 17.3% versus 6.3%, respectively (Table 2).

Former and newly AOs generally showed intermediate prevalence levels. In several cases, such as hazardous alcohol use and economic hardship, both newly and persistent affected others differed significantly from never affected respondents, indicating that both recent and sustained exposure were associated with an elevated prevalence of these outcomes (Table 2).

### Longitudinal patterns and within-group changes across AO trajectories

**Fig. 1:**
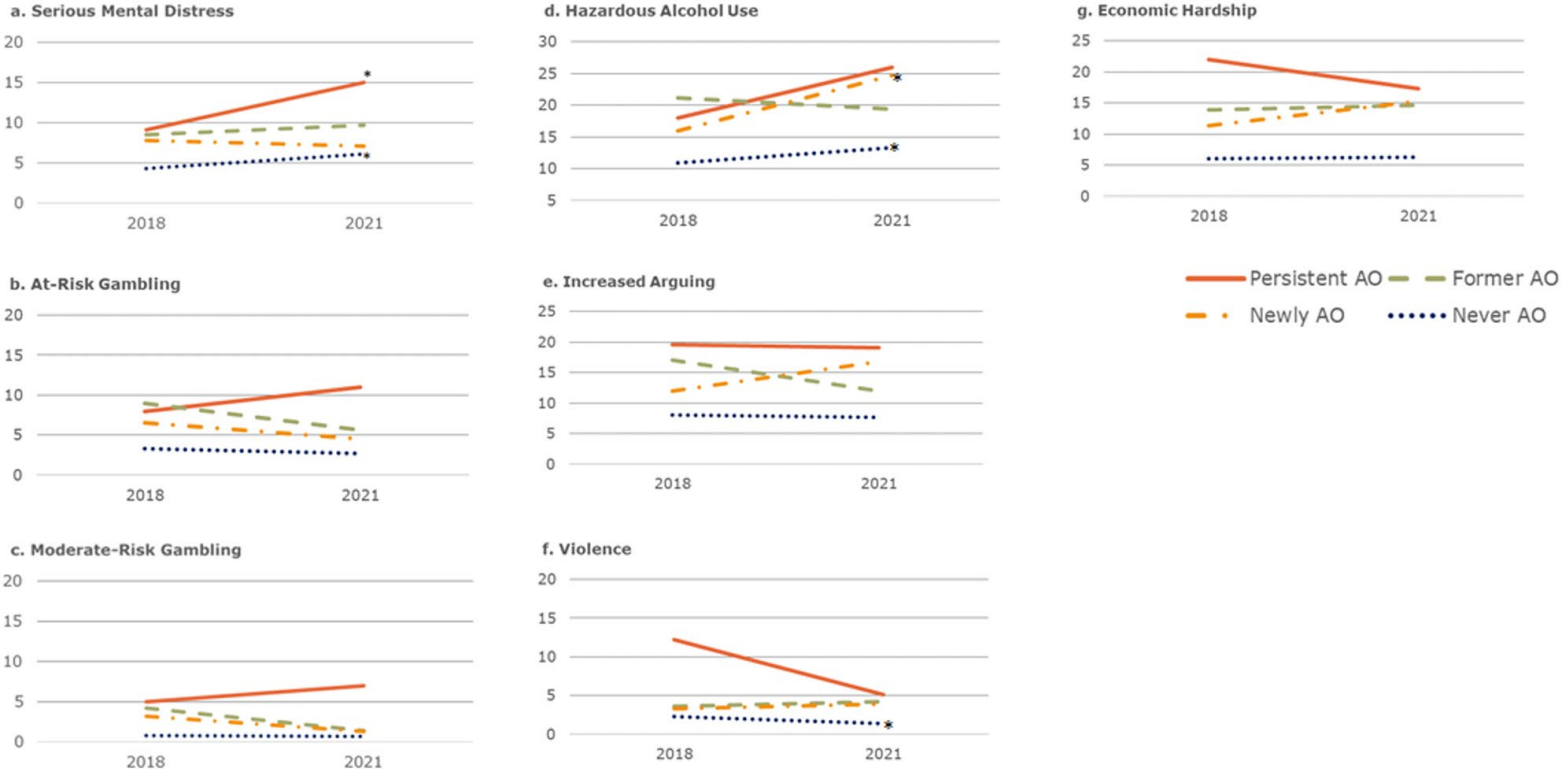
**a**-**g** Prevalence of past year outcomes in 2018 and 2021, stratified by longitudinal AO status (persistent, former, newly, and never AO). Significant within-group changes marked with *p = < 0.05. **a** serious mental distress (K6 ≥ 13); **b** at‑risk gambling (PGSI ≥ 1); **c** moderate-risk gambling (PGSI ≥ 3); **d** hazardous alcohol use (AUDIT‑C ≥ 5 for women, 6 for men); **e** increased arguing with close others; **f** exposure to physical violence or threats of violence; **g** economic hardship (difficulties managing everyday expenses)

Figure 1a-g illustrate changes in adverse outcomes between 2018 and 2021 across AO trajectories. Overall, persistent AOs consistently showed the highest prevalence at both time points, with stable or increasing trends across most of the outcomes. Newly AOs showed mixed patterns, with increasing trends for some outcomes, primarily hazardous alcohol use, but declining trends for gambling severity and mental distress. Never AOs remained comparatively stable across the outcomes.

Within-group analyses indicated that serious mental distress increased significantly among persistent AOs, and alcohol use increased significantly among newly AOs (supplementary Table S1). Changes in relational and economic outcomes were not statistically significant within any trajectory.

### Associations between affected‑other trajectories and adverse outcomes

Multivariable logistic regression analyses further demonstrated that affected-other trajectories were differentially associated with adverse outcomes in 2021, after adjusting for sex, age, and moderate-risk gambling, where applicable (own moderate-risk gambling was not included as a covariate in gambling-related outcomes) (Table 3).

**Table 3:** Odds ratios (ORs) and 95% confidence intervals (CIs) for past–year outcomes among former, newly, and persistent affected others compared with never affected respondents. Adjusted models included sex, age, and own moderate-risk gambling (PGSI ≥ 3); for gambling, PGSI was not included as a covariate

		Affected Other Status	Crude Model		Adjusted Model	
		(ref Never AO)	OR (CI)	*p*–value	OR (CI)	*p*–value
Mental health Outcomes	Serious Mental Distress	Former	1.65 (0.93–2.93)	0.089	1.53 (0.85–2.73)	0.155
		Newly	1.18 (0.62–2.22)	0.615	0.99 (0.52–1.89)	0.978
		Persistent	**2.70** (1.52–4.80)	< 0.001	**2.18** (1.20–3.95)	0.011
	Suicidal Ideation	Former	1.90 (0.85–4.22)	0.117	1.59 (0.71–3.58)	0.261
		Newly	1.77 (0.80–3.92)	0.163	1.30 (0.57–2.93)	0.532
		Persistent	**4.58** (2.33–9.0)	< 0.001	**2.75** (1.33–5.68)	0.006
	At-Risk Gambling	Former	2.09 (0.99–4.45)	0.055	**2.25** (1.04–4.85)	0.039
		Newly	1.70 (0.76–3.76)	0.194	1.58 (0.70–3.57)	0.269
		Persistent	**4.40** (2.25–8.62)	< 0.001	**4.08** (2.03–8.17)	< 0.001
	Moderate-Risk Gambling	Former	2.11 (0.48–9.28)	0.322	2.24 (0.50–10.0)	0.231
		Newly	1.97 (0.45–8.66)	0.368	2.03 (0.45–9.08)	0.356
		Persistent	**11.29** (4.54–28.11)	< 0.001	**10.34** (4.02–26.68)	< 0.001
	Hazardous Alcohol Use	Former	**1.57** (1.02–2.41)	0.039	1.45 (0.94–2.24)	0.096
		Newly	**2.13** (1.45–3.13)	< 0.001	**1.67** (1.13–2.48)	0.011
		Persistent	**2.29** (1.22–3.63)	< 0.001	**1.84** (1.14–2.97)	0.012
Relational outcomes	Increased Arguing	Former	1.64 (0.95–2.84)	0.74	1.42 (0.81–2.46)	0.218
		Newly	**2.43** (1.52–3.86)	< 0.001	**2.08** (1.28–3.37)	0.003
		Persistent	**2.85** (1.67–4.87)	< 0.001	**2.24** (1.28–3.92)	0.005
	Violence	Former	**3.14** (1.30–7.63)	0.011	**2.83** (1.16–6.90)	0.023
		Newly	**2.91** (1.20–7.05)	0.018	2.36 (0.95–5.83)	0.064
		Persistent	**3.82** (1.46–9.99)	0.006	**2.97** (1.10–8.03)	0.032
Economic outcomes	Economic Hardship	Former	**2.54** (1.56–4.16)	< 0.001	**2.17** (1.31–3.57)	0.002
		Newly	**2.70** (1.68–4.33)	< 0.001	**2.35** (1.45–3.83)	< 0.001
		Persistent	**3.13** (1.81–5.41)	< 0.001	**2.21** (1.25–3.91)	0.006

Note. Bold values indicate statistically significant associations (*p* < .05)

In the adjusted models, persistent AOs showed significantly higher odds than never AOs across all outcomes examined. The strongest associations were observed for moderate-risk gambling and suicidal ideation: persistent AOs had over tenfold higher odds of moderate-risk gambling (AOR = 10.34, 95% CI 4.02–26.68) and nearly threefold higher odds of suicidal ideation (AOR = 2.75, 95% CI 1.33–5.68) than never AOs. Substantially elevated odds were also observed for at-risk gambling, serious mental distress, hazardous alcohol use, increased arguing, exposure to violence, and economic hardship (Table 3).

For former AOs, most associations were attenuated after adjustment, although significantly elevated odds remained for at-risk gambling (AOR = 2.25, 95% CI 1.04–4.85), exposure to violence (AOR = 2.83, 95% CI 1.16–6.90), and economic hardship (AOR = 2.17, 95% CI 1.31–3.57).

Newly AOs showed significantly higher odds of hazardous alcohol use (AOR = 1.67, 95% CI 1.13–2.24), increased arguing (AOR = 2.08, 95% CI 1.28–3.37), and economic hardship (AOR = 2.35, 95% CI 1.45–3.83) than never AOs.

### Summary of results

Across the analyses, persistent AO exposure emerged as the most consistent and robust correlate of adverse outcomes. Cross-sectional analyses demonstrated substantially higher prevalence of mental, relational, and economic outcomes among persistent affected others (Table 2), longitudinal analyses showed worsening mental distress over time within this group (Table S1 in the Supplementary Material), and regression models confirmed elevated adjusted odds across multiple outcomes (Table 3). Former AOs generally showed attenuated risks compared with persistent AOs, although elevated odds persisted for selected outcomes, including violence and economic hardship. Newly AOs showed a more selective pattern, with elevated odds primarily for alcohol use, relational conflict, and economic hardship, suggesting that even recent exposure may be associated with adverse outcomes in specific domains.

## Discussion

This longitudinal population-based study examined health (including serious mental distress, suicidal ideation, hazardous alcohol use, and own gambling behaviour), relational (arguing, exposure to violence), and economic outcomes across four AO trajectories over a three-year period. The findings consistently show that persistent exposure to perceived problem gambling in one’s social environment is associated with the most adverse outcome profiles, whereas former AO status generally corresponds to attenuated risks. These results extend previous cross-sectional research by demonstrating that the duration and continuity of exposure are important for understanding the health burden among affected others.

The most robust finding was the consistently elevated odds of adverse outcomes among persistent AOs across all domains examined, including serious mental distress, suicidal ideation, at-risk gambling, hazardous alcohol use, relational conflict, and economic hardship. These associations remained after adjusting for sex, age, and moderate-risk gambling, suggesting that persistent exposure to a close other’s gambling problems is independently associated with a broad range of adverse outcomes. From a theoretical perspective, this is consistent with stress and coping models, which propose that chronic exposure to a significant other’s problematic behaviour depletes coping resources over time, leading to accumulated strain across multiple life domains [17, 24]. The longitudinal within-group analyses further supported this interpretation, showing a significant increase in serious mental distress among persistent AOs between 2018 and 2021, suggesting that mental health consequences may worsen with continued exposure rather than stabilising over time.

Former AOs generally showed intermediate outcome profiles, with most associations attenuated after adjustments. This pattern is broadly consistent with the findings of Svensson et al. [5], who found that individuals no longer classified as AOs at follow-up reported better mental health and fewer relational problems than those who remained AOs. However, the present findings also indicate that not all consequences resolve after the cessation of exposure. Economic hardship remained significantly elevated among former AOs even after adjustment, suggesting that financial consequences may be more persistent than psychological or relational harm. This is consistent with previous research showing that financial well-being among affected others tends not to improve over time to the same extent as among non-affected individuals, potentially offsetting expected improvements in economic circumstances [7]. These findings underline the importance of distinguishing between harm domains when examining recovery trajectories among affected others.

Newly AOs showed a significant increase in hazardous alcohol use between 2018 and 2021, and elevated odds of alcohol use, increased arguing, and economic hardship compared with never AOs. The alcohol finding is particularly notable, as it may reflect early coping responses to a stressful social situation rather than a pre-existing vulnerability, given that these individuals were not classified as AOs at the baseline. This tentative interpretation warrants further investigation but aligns with previous cross-sectional findings linking AO status to elevated alcohol use [4, 5, 25, 26]. Alternatively, elevated alcohol use among newly AOs may partly reflect shared social environments in which both gambling problems and risky alcohol use are more prevalent, rather than a direct response to exposure. This alternative interpretation is consistent with findings from an Australian population-representative study, in which the association between AO status and binge drinking was significant in models adjusting for basic demographics but attenuated to non-significance once a broader range of mental health characteristics was accounted for [10], suggesting that shared vulnerability factors may partly explain the association.

Across all analyses, never AOs served as a stable reference group with a consistently lower prevalence of adverse outcomes, consistent with the trajectory classification capturing meaningful differences in exposure. The graded pattern observed across trajectories, with never AOs showing the lowest, former and newly AOs showing intermediate, and persistent AOs showing the highest levels of adverse outcomes, suggests a dose-response-like relationship between exposure duration and harm burden. However, this graded pattern does not by itself rule out selection effects, such as pre-existing vulnerability factors that could independently increase both the likelihood of knowing someone with gambling problems and the risk of adverse outcomes, and the cross-sectional nature of most comparisons precludes a causal interpretation.

Notwithstanding these caveats, the findings may also have relevance for how support is organised for affected others. Within the Stress-Strain-Coping-Support framework, information and understanding, social support, communication, and coping skills have been identified as central mechanisms through which strain on close others can be addressed [27]. Given that former, newly, and persistent AOs showed distinct patterns of outcomes in the present study, screening procedures that take exposure history into account, rather than exposure status alone, may help identify individuals with different support needs, consistent with recommendations for tailored rather than uniform intervention approaches [27]. This may be particularly important given that affected others remain markedly underrepresented in treatment relative to their numbers, with a lack of service awareness and stigma frequently cited as barriers to help-seeking [28].

### Strengths and limitations

The longitudinal design enables the examination of temporal patterns, which remain rare in research on affected others. The use of a large population-based sample enhances external validity and allows comparisons across well-defined exposure trajectories. Moreover, the inclusion of multiple outcome domains, including health, relational, and economic outcomes, provides a comprehensive assessment of the broader impact of gambling problems on affected others. Finally, the conceptualisation of AO status as a dynamic condition represents a methodological strength, allowing for differentiation between persistent, former, and newly affected individuals, which extends previous research that has predominantly relied on cross-sectional design.

At the same time, the longitudinal sample was restricted to respondents participating in both waves, which may limit generalisability. AO status was measured using a single broad item capturing exposure rather than directly experienced harm, which means that the AO groups likely included heterogeneous situations varying in relational proximity, contact frequency, and actual harm experienced. Furthermore, although the models adjusted for sex, age, and moderate-risk gambling, other unmeasured factors, such as pre-existing psychological vulnerability, socioeconomic circumstances, or family history of substance use problems, could not be accounted for. It is therefore possible that some of the observed associations partly reflect selection, whereby individuals with pre-existing vulnerabilities are more likely to know someone with gambling problems, rather than a direct consequence of that exposure. Additionally, the outcome measures did not specifically ask whether the reported relational, economic, or health difficulties were linked to the person with gambling problems, meaning that some of the reported outcomes may reflect circumstances unrelated to that specific relationship. Future studies should therefore aim to distinguish exposure from directly experienced harm, incorporate a broader range of baseline covariates, and consider outcome measures that explicitly ask respondents to attribute the difficulty to the affected relationship.

In addition, within-group analyses were limited by relatively small sample sizes in the former, newly, and persistent AO groups, which reduced the statistical power to detect significant changes over time. Finally, although the design was longitudinal, the use of only two measurement points limited the ability to capture more complex trajectories and the underlying mechanisms of change. As such, this study should be regarded as an early, hypothesis-generating investigation of AO trajectories, providing a basis for more targeted future research rather than a definitive test of causal pathways.

## Conclusion

This study provides longitudinal population-based evidence that the duration and continuity of exposure to perceived gambling problems in one’s social environment are linked to a range of adverse mental, relational, and economic outcomes. Persistent AO status was consistently associated with the most adverse outcome profiles, while former AOs generally showed attenuated risks, with the exception of economic hardship, which remained elevated. These findings suggest that AO status may be better conceptualised as a dynamic condition than a fixed one, and that sustained as well as past exposure may have lasting consequences. Therefore, prevention and support efforts should consider both the duration and timing of exposure when identifying individuals at a heightened risk. In addition, given the consistent associations observed between AO status and hazardous alcohol use, relational conflict, and economic hardship, individuals presenting with these difficulties in clinical or support settings may also benefit from being asked about exposure to a close other’s gambling problems.

## Supplementary information

Supplementary Material 1.

## Data Availability

The data that support the findings of this study are available from Statistics Sweden (SCB). Access to the data is subject to ethical approval and approval from the Swedish Public Health Agency, which is the data controller. Due to legal and ethical restrictions, the data are not publicly available but can be accessed by qualified researchers upon reasonable request and approval from the relevant authorities.

## Abbreviations

AO: Affected other
AUDIT: C–Alcohol use disorders identification test–consumption
K6: Kessler psychological distress scale
PGSI: Problem gambling severity index
Swelogs: Swedish longitudinal gambling study
