# Supplementary material for: Problem gambling and affected others: health, relational and economic outcomes across longitudinal trajectories in a population-based Swedish study
Source: BMC Public Health. 2026 Aug 10;26:2318. doi: 10.1186/s12889-026-28778-z (PMC13459228; doi:10.1186/s12889-026-28778-z)
Supplement: Supplementary file 1 [file 12889_2026_28778_MOESM1_ESM.docx]

**Table 1S.** Within-group changes in adverse outcomes between 2018 and 2021 by affected-other (AO) trajectory.

| Outcome | AO trajectory | N (pairs) | Test | Statistic | p |
| --- | --- | --- | --- | --- | --- |
| Mental distress (index, 0-30) | Never | 2 258 | Wilcoxon | Z = −6.08* | < .001 |
|  | Former | 138 |  | Z = −0.48 | .629 |
|  | Newly | 152 |  | Z = −1.44 | .151 |
|  | Persistent | 96 |  | Z = −2.99* | .003 |
| Gambling (index, 0-27) | Never | 2 416 | Wilcoxon | Z = −1.38 | .169 |
|  | Former | 144 |  | Z = −1.83 | .067 |
|  | Newly | 154 |  | Z = −0.88 | .379 |
|  | Persistent | 100 |  | Z = −0.41 | .680 |
| Alcohol Use (index, 0-12) | Never | 2 380 | Wilcoxon | Z = −3.60* | < .001 |
|  | Former | 142 |  | Z = −1.09 | .277 |
|  | Newly | 151 |  | Z = −2.70* | .007 |
| Increased arguing (binary) | Never | 2 249 | McNemar | χ² = 0.31 | .577 |
|  | Former | 130 |  | χ² = 2.37 | .124 |
|  | Newly | 140 |  | χ² = 0.83 | .361 |
|  | Persistent | 92 |  | Exact | 1.000 |
| Violence (binary) | Never | 2 376 | McNemar | Χ² = 7.78* | .005 |
|  | Former | 138 |  | Exact | 1.000 |
|  | Newly | 150 |  | Exact | 1.000 |
|  | Persistent | 97 |  | Exact | .092 |
| Economic hardship (binary) | Never | 2 359 | McNemar | Exact | 1.000 |
|  | Former | 142 |  | Exact | 1.000 |
|  | Newly | 147 |  | Exact | .383 |
|  | Persistent | 98 |  | Exact | .332 |

*Note. Within-group analyses compare outcomes in 2018 and 2021 within each affected-other (AO) trajectory (never AO, former AO, newly affected AO, and persistent AO). Index outcomes (alcohol use, gambling, and mental distress) were analyzed using Wilcoxon signed-rank tests. Binary outcomes (arguments, exposure to violence, and economic hardship) were analyzed using McNemar’s test. Asymptotic χ² statistics are reported when applicable; otherwise, exact p-values based on the binomial distribution are presented. Analyses are based on paired observations only; sample sizes therefore vary across outcomes. The sign of the Wilcoxon Z statistic depends on the ordering of differences and should not be interpreted as indicating direction of change.*
